# From Prediction to Precision: Explainable AI-Driven Insights for Targeted Treatment in Equine Colic

**DOI:** 10.3390/ani15020126

**Published:** 2025-01-08

**Authors:** Bekir Cetintav, Ahmet Yalcin

**Affiliations:** 1 Department of Biostatistics, Veterinary Faculty, Burdur Mehmet Akif Ersoy University, 15030 Burdur Merkez, Turkey; 2 Institute of Science, Burdur Mehmet Akif Ersoy University, 15030 Burdur Merkez, Turkey; ahmtylcinn15@gmail.com

**Keywords:** equine colic, explainable artificial intelligence (XAI), machine learning in veterinary medicine, SHAP, precision animal health management, targeted veterinary medicine

## Abstract

Colic is a leading cause of mortality in horses, demanding precise and timely interventions. This study integrates machine learning and explainable artificial intelligence (XAI) to predict survival outcomes in horses with colic, using clinical, procedural, and diagnostic data. Random forest and XGBoost emerged as top-performing models, achieving F1 scores of 85.9% and 86.1%, respectively. SHAP (Shapley additive explanations) was employed to provide interpretable insights, offering both global and local explanations for model predictions. The analysis revealed that key features, such as pulse rate, lesion type, and total protein levels, significantly influenced survival likelihood. Local interpretations highlighted the unique contribution of clinical factors to individual cases, enabling personalized insights that guide targeted treatment strategies. These tailored predictions empower veterinarians to prioritize interventions based on the specific conditions of each horse, moving beyond generalized care protocols. By combining predictive accuracy with interpretability, this study advances precision veterinary medicine, enhancing outcomes for equine colic cases and setting a benchmark for future applications of AI in animal health.

## Simple Summary

Colic is a critical health issue for horses, often requiring immediate and precise intervention to improve survival rates. This study uses machine learning and explainable artificial intelligence (XAI) techniques to predict the likelihood of survival for horses affected by colic. By analyzing clinical, procedural, and diagnostic data, the model identifies key factors that influence outcomes, such as pulse rate, lesion type, and protein levels. The use of SHAP (Shapley additive explanations) ensures transparency and enables targeted treatments, improving the welfare of horses and advancing precision veterinary care. Such innovations highlight the importance of combining technology and veterinary science for better animal health management.

## 1. Introduction

Colic remains one of the most significant health concerns in equine medicine due to its complex etiology, high mortality rates, and frequent complications requiring prompt diagnosis and intervention. Defined as acute abdominal pain, colic encompasses a range of gastrointestinal and abdominal disorders that can result in severe systemic effects if untreated [1,2]. Despite advancements in veterinary care, colic persists as a leading cause of morbidity and mortality among horses, with surgical intervention often required in 10–20% of cases [3]. The prognoses and outcomes of colic cases are highly variable, influenced by factors such as lesion type, age, and systemic health parameters [4].

The success of colic management depends on several preoperative, intraoperative, and postoperative factors. Preoperative indicators, including age, the duration of clinical signs, and systemic health assessments (e.g., packed cell volume and mucous membrane color), are critical for predicting survival [5,6]. Intraoperatively, the type and severity of lesions, such as strangulating versus non-strangulating obstructions, and the surgical techniques employed significantly impact outcomes [4]. Postoperative complications like ileus, septic peritonitis, and adhesions remain major challenges, often dictating long-term survival and quality of life for affected horses [1,6]. Parameters like packed cell volume and total protein levels provide further insight into dehydration and systemic compromise, which are frequently associated with severe colic cases. Advanced diagnostic techniques, such as abdominocentesis, offer valuable information about abdominal conditions, helping clinicians to identify surgical lesions and predict outcomes [7].

Predictive models have emerged as a transformative tool in equine treatment and emergencies, particularly in managing complex and critical conditions like colic. These models leverage machine learning (ML) algorithms to analyze clinical, historical, and procedural data, enabling accurate predictions of outcomes such as survivability likelihood and the need for surgical intervention [3,8,9]. In equine medicine, predictive models have been used to analyze pre- and post-operative mortality risks in colic surgeries, with findings highlighting the importance of easily accessible variables like lesion type and patient age in improving clinical decision-making [3]. The effectiveness of ML extends to complex diagnostic tasks, such as identifying risk factors for brucellosis in dairy cattle, where methods like classification and regression trees (CART) have outperformed traditional logistic regression models in accuracy and interpretability [10]. Furthermore, ML has been applied to predict lameness in dairy cows, providing actionable insights for precision farming by utilizing conformation traits and management data to anticipate disease risks [11].

While prediction accuracy is crucial, the true value of ML in equine emergencies lies in its ability to provide actionable insights. The integration of explainable artificial intelligence (XAI) techniques, such as SHAP (Shapley additive explanations), transforms these “black-box” models into interpretable frameworks. Beyond merely predicting outcomes, these insights can guide targeted treatments, optimize resource allocation, and ultimately improve patient outcomes. For instance, XAI frameworks have been applied in heart disease prediction to enhance clinician trust by clarifying the influence of features such as cholesterol levels and exercise-induced angina [12]. Similarly, in the context of Alzheimer’s disease, SHAP has enabled a deeper understanding of the clinical importance of biomarkers, fostering alignment between model outputs and clinical expertise [13]. SHAP was utilized to identify key factors like doctor’s recommendations in vaccine hesitancy prediction, offering actionable insights for public health interventions [14]

In this study, we present a machine learning-based approach to predict survivability in horses affected by colic, focusing on integrating clinical, procedural, and diagnostic parameters into robust predictive models. By leveraging supervised learning algorithms and explainable AI (XAI) techniques, we aim to not only achieve high predictive accuracy but also provide interpretable insights into the key factors influencing outcomes. Our methodology also emphasizes the importance of transparency in AI-driven predictions, enabling veterinarians to better understand the rationale behind the models’ decisions. This interpretability fosters trust and facilitates targeted treatment strategies, ensuring that the models serve as actionable tools in clinical settings. Ultimately, our approach bridges the gap between advanced predictive modeling and practical veterinary applications, contributing to improved outcomes in equine emergency care.

## 2. Materials and Methods

**Figure 1:**
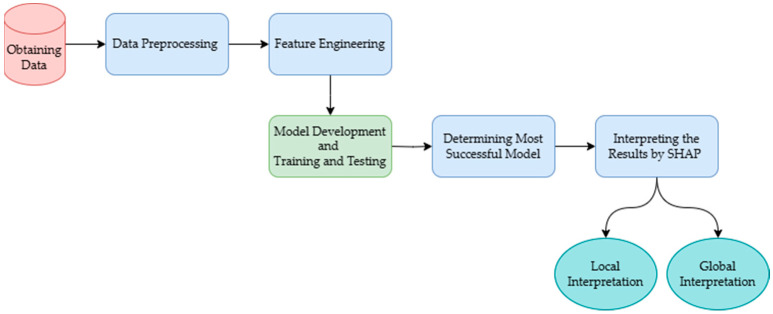
Integrated framework to predict horse survival and provide post-explanations.

In this study, we build an integrated framework to predict horse survival and provide post-explanations for the predictions (Figure 1). The process begins with acquiring a dataset containing clinical and physiological information relevant to equine health and survival outcomes. The data are then cleaned and preprocessed to handle inconsistencies, missing values, or outliers, ensuring high-quality data for model training. Feature engineering is employed to enhance the predictive power by transforming or creating new variables. Subsequently, various machine learning models are developed and trained on the processed data to predict the likelihood of horse survival. SHAP (Shapley additive explanations) is utilized to generate both local and global explanations of the models. Local explanations provide insights into individual predictions, while global explanations offer transparency and interpretability by illustrating the impact of each feature on predictions across the dataset.

### 2.1. Data Collection and Preprocessing

The dataset used in this study was taken from a well-known study [15], which provides comprehensive information on the colic status of horses. The dataset was retrieved from the UCI Machine Learning Repository (Horse Colic Dataset), a platform that provides open access to datasets under the Creative Commons Attribution 4.0 International (CC BY 4.0) license. It comprises 299 records of horse health indicators, encompassing both clinical and physiological features such as rectal temperature, pulse, respiratory rate, mucous membrane status, and packed cell volume (Appendix A). The target variable indicates the survival outcome of horses: “lived”, “died”, or “euthanized” (in our study, “euthanized” cases were grouped under “died”, to ensure binary classification).

Missing data were imputed using classical techniques: the mode for categorical variables and the mean or median for numerical ones, depending on their distribution. Feature engineering included label encoding for categorical variables, removal of irrelevant columns, and decoding of “Lesion” features into meaningful categories. To address class imbalance in the target variable, the synthetic minority oversampling technique (SMOTE) was applied, generating synthetic samples for the minority class (“died”) and balancing the dataset. Detailed descriptions of these processes are provided in Appendix B.

### 2.2. Prediction Model Development and Evaluation

In this study, several machine learning models were employed to predict horse survival outcomes, including decision trees, support vector machines (SVM), random forests, and gradient boosting algorithms (Table 1). Each model was selected based on its ability to handle categorical and numerical features while addressing the complexities of the dataset. For instance, decision trees and random forests were used due to their inherent feature importance and interpretability, while SVM and gradient boosting provided robustness against class imbalance and non-linear relationships.

The models were trained and evaluated using a balanced dataset, achieved through the SMOTE. This ensured adequate representation of minority class samples (“died”), improving the models’ ability to generalize effectively. Model evaluation was performed using performance metrics such as accuracy, precision, recall, and F1 score. In this study, Recall and F1 Score were prioritized as key performance metrics due to the critical importance of accurately identifying high-risk cases (“died”) and balancing precision and recall in the presence of class imbalance. These metrics ensure robust and reliable predictions, particularly for the minority class, which is vital in survival analysis. Detailed descriptions of all metrics are provided in Appendix B.

### 2.3. Post-Explainability Techniques

Explainable artificial intelligence (XAI) encompasses a range of methodologies aimed at making the outputs of artificial intelligence systems interpretable to humans. These techniques enhance the understanding of AI models by elucidating the underlying motivations and processes used to generate predictions. Among the prominent algorithms in this domain is SHAP (Shapley additive explanations), introduced by Lundberg and Lee in 2017 [32]. SHAP employs a game-theoretic framework to quantify the contribution of each feature to a model’s prediction, providing insights into the relative importance of individual attributes. Unlike traditional feature importance measures, SHAP enables a granular analysis of each attribute’s influence on classification outcomes.

In this study, we employed SHAP to interpret the predictions of the most accurate machine learning model. SHAP values were utilized to provide both global and local explanations, facilitating a comprehensive understanding of the model’s behavior. At the global level, SHAP elucidates how features collectively influence predictions across the entire dataset, while at the local level, it highlights the contribution of specific features to individual predictions. This dual approach ensures that the model’s decision-making process is both transparent and interpretable, aligning with the principles of XAI.

#### 2.3.1. Local Explanation

SHAP values, which illustrate the precise contribution of each trait to the adoption chances of a single pet, offer reasons for individual predictions at the local level. This approach is particularly valuable in interpreting case-by-case scenarios, as SHAP can reveal, for instance, how elevated packed cell volume or abnormal mucous membrane color influences the survival probability of a specific horse. These localized insights enable veterinarians to understand why the model assigned a particular survival probability to an individual case. Such transparency facilitates more personalized decision-making, allowing practitioners to prioritize interventions tailored to the unique conditions of each horse. The local SHAP value for a feature i for an instance x is given by the following equation:

(1) $$\phi_{j}\left(i\right)=\sum_{S\;\subseteq N\backslash \backslash \left\{i\right\}}\frac{\left|S\right|!\left(\left|N\right|-\left|S\right|-1\right)!}{\left|N\right|!}\;\;\left(f_{x}\left(S\cup \left\{i\right\}\right)-f_{x}\left(S\right)\right)$$

where N is the set of all features, S is a subset of features that does not include i, $\left|S\right|$ is the number of features in subset S, $f_{x}\left(S\right)$ is the model prediction using only the features in subset S and $\left(f_{x}\left(S\cup \left\{i\right\}\right)-f_{x}\left(S\right)\right)\;$ represents the marginal contribution of feature i when it is added to subset S.

This formula computes the weighted average of feature i’s contribution across all possible feature subsets, producing a SHAP value that reflects the feature’s impact on the specific prediction.

#### 2.3.2. Global Explanation

When it comes to the overall significance of each characteristic in the model’s decision-making process, SHAP offers insightful information for a general perspective. For instance, in our horse survival prediction model, SHAP analysis revealed that features such as packed cell volume, mucous membrane color, and rectal temperature were significant drivers in predicting survival likelihood across the dataset. By aggregating SHAP values across all data points, we gain a global perspective on which features exert the most substantial influence, enabling us to identify key patterns and tendencies in survival outcomes. This global understanding is critical for veterinarians and equine caregivers, as it highlights which general factors, such as physiological indicators and clinical symptoms, are most impactful in determining survival probabilities. Such insights inform more effective management strategies and interventions to improve overall survival outcomes.

(2) $${\hat{\phi }}_{j}=\frac{1}{M}\;\sum_{m=1}^{M}\left(\;\hat{f}\left(x_{+j}^{m}\right)-\hat{f}\left(x_{-j}^{m}\right)\right)\;\;\;\;\;\;\;\;\;\;\;\;\;$$

- In Equation (2), M is the number of iterations, x is the sample of interest, j is the attribute index and f is the machine learning model. ‘$\hat{f}\left(x_{+j}^{m}\right)$’ is the prediction for x, but with the exception of the corresponding value of attribute j, a random number of attribute values were replaced with attribute values from random z data points.
- The procedure must be repeated for each feature to obtain all Shapley values (Equations (1) and (2) are taken from the main study of [17]).

### 2.4. Used Technologies

This study utilized Python v.3.9 and its libraries to support data preprocessing, model development, evaluation, and explainability. Libraries such as *pandas* and *numpy* were employed for efficient data manipulation and numerical computations, while *matplotlib* and *seaborn* facilitated data visualization. For preprocessing, *scikit-learn* was used for label encoding and implementing machine learning models, and *imblearn* was applied to address class imbalance through the SMOTE. Gradient boosting models were developed using specialized boosting libraries, while the *SHAP* library provided both local and global explanations of model predictions.

## 3. Results

To evaluate the predictive performance of the models, accuracy, recall, precision, and F1 score were used as key metrics (Table 2). Ensemble-based methods consistently outperformed simpler algorithms, demonstrating their ability to handle the dataset’s complexity effectively.

Among the tested models, Random Forest achieved the highest accuracy performance, with an accuracy of 86.1%, recall of 85.9%, and F1 score of 85.9%. XGBoost followed closely with similar accuracy (86%), but higher precision (86.2%), recall of 86.0%, and F1 score of 86.0%, making it the top performer. Other ensemble models, such as HistGradientBoost, LightGBM (LGBM), and AdaBoost, also demonstrated strong results, while simpler models like K-Nearest Neighbors (KNN) and Support Vector Machine (SVM) showed lower performance, reflecting their limitations with this dataset.

For the remainder of the study, XGBoost was selected as the primary model due to its robust performance, particularly in terms of Recall and F1 Score, which were critical for accurately identifying high-risk cases (“died”). These metrics align with the study’s objective of ensuring reliable predictions for the minority class.

### 3.1. SHAP Local Interpretation

Two horses were chosen from the dataset as samples for local interpretation. Care was taken to ensure that the target variable of the selected samples was “died”. The examples’ Shapley values were computed and displayed using a waterfall plot. By displaying the cumulative effect of each feature on the baseline prediction, a waterfall plot illustrates how distinct elements in a machine learning model contribute to a particular prediction. Each feature’s contribution is shown as a bar, with red bars denoting positive contributions (raising the forecast) and blue bars denoting negative contributions (lowering the prediction), starting with the baseline value, which is the model’s average. Each bar’s length indicates the impact’s magnitude; longer bars indicate larger contributions [33].

**Figure 2:**
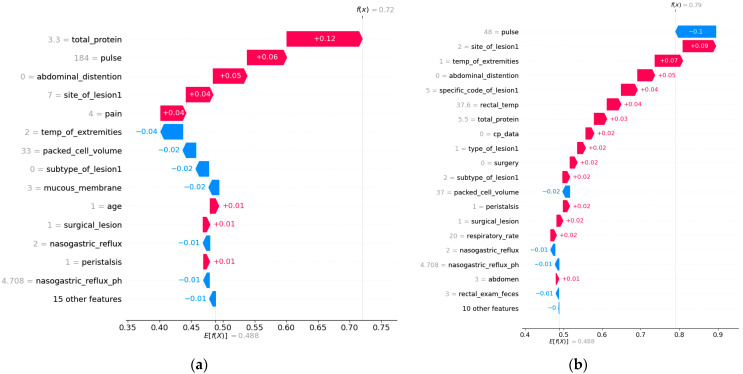
SHAP waterfall plots for local interpretation. (**a**) Waterfall plot for Horse 1 showing the contribution of individual features to the “died” prediction. (**b**) Waterfall plot for Horse 2 showing the contribution of individual features to the “died” prediction.

The two SHAP waterfall plots (Figure 2) illustrate how different features contribute to the “died” prediction for two separate horses, highlighting the nuanced and individualized nature of the model’s decision-making process. Despite both predictions indicating a high probability of death, the dominant contributing features vary between the cases, reflecting the complex interplay of clinical indicators in equine health.

In the first plot (Figure 2a), **total protein (+0.12)**, **pulse (+0.06)**, and **abdominal distention (+0.05)** are the strongest contributors to the prediction. These features emphasize metabolic disturbances, cardiovascular distress, and gastrointestinal complications as the primary drivers of the horse’s poor outcome. Negative contributions, such as normal **packed cell volume (−0.02)** and **mucous membrane (−0.02)**, provided some stability but were insufficient to counteract the dominant positive factors.

In the second plot (Figure 2b), **pulse (−0.1)** and **site of lesion 1 (+0.07)** were the top contributors, with abnormal extremity temperature (+0.05) playing a significant role. This case highlights cardiovascular issues and lesion-related severity as critical factors, with other features like **specific code of lesion 1 (+0.04)** and **rectal temperature (+0.04)** reinforcing the prediction. Other negative contributions, such as pulse (−0.1), packed cell volume (−0.02), and nasogastric reflux (−0.01), again played a stabilizing role but could not eliminate the individual from the died class.

Both cases reveal common themes, such as cardiovascular and lesion-related indicators being key predictors of death, but the relative importance of features differs between the two examples. This variability underscores the value of SHAP in providing personalized insights, enabling veterinarians to prioritize interventions based on the specific conditions of each horse.

### 3.2. SHAP Global Interpretation

**Figure 3:**
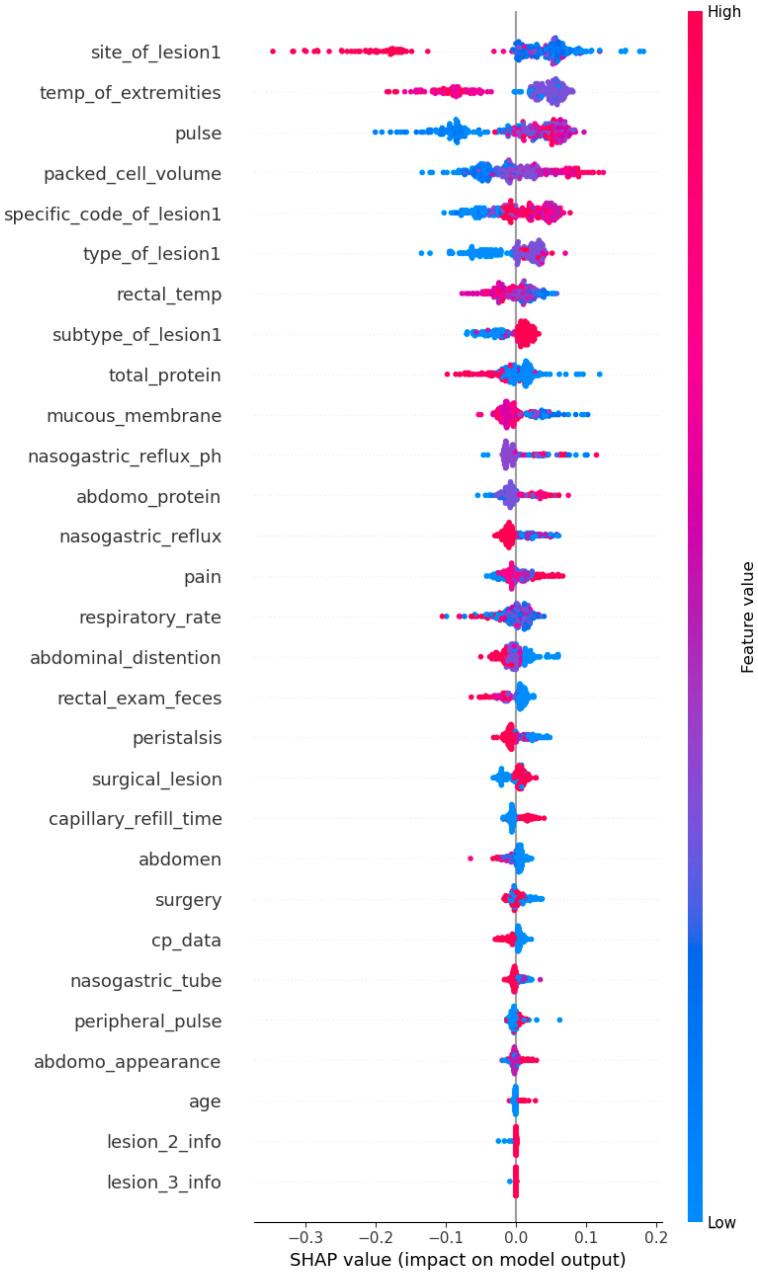
SHAP summary plot for global interpretation.

SHAP (Shapley additive explanations) values were computed and displayed in the summary plot below (Figure 3) in order to explain the XGBoost’s prediction performance throughout the full dataset. This global interpretation sheds light on how each feature affects the likelihood of pet adoption forecasts made by the algorithm. The summary graphic combines feature relevance and feature effects. Each point on the summary plot represents a Shapley value for a feature and an instance. The position on the x-axis is determined by the Shapley value, while the position on the y-axis is determined by the feature. The hue represents the feature’s contribution, ranging from low to high. Because overlapping points are jittered in the y-axis direction, we can observe the distribution of Shapley values for each feature. The features are arranged according to their significance [33].

The plot (Figure 3) illustrates the impact of each feature on the model’s predictions across the entire dataset. Features are ranked by their importance, with site_of_lesion1, temp_of_extremities, and pulse being the most influential. The color gradient represents the feature values, where red indicates high values and blue indicates low values, showing their respective contributions to the prediction outcomes. The alignment of these findings with clinical knowledge underscores the model’s validity and utility.

## 4. Discussion

This study highlights the transformative potential of explainable artificial intelligence (XAI) in advancing equine colic management, with personalized insights derived through SHAP (Shapley additive explanations) emerging as the most impactful contribution. By offering interpretable, case-specific explanations for model predictions, SHAP empowers veterinarians to tailor interventions based on the unique clinical profiles of individual horses, moving beyond generalized treatment protocols and paving the way for precision veterinary medicine.

In the first case analyzed (Figure 2a), features such as elevated total protein, increased pulse, and abdominal distention were identified as dominant contributors to the prediction of a poor outcome. Based on these insights, a veterinarian could prioritize interventions such as aggressive fluid therapy to address dehydration, cardiovascular support to stabilize pulse, and diagnostic imaging to assess abdominal health. Similarly, in the second case (Figure 2b), critical contributors included the site of lesion and abnormal extremity temperature, suggesting the need for immediate surgical exploration and systemic stabilization. These scenarios align with previous studies emphasizing the role of specific clinical indicators in predicting survival [5,6].

Globally, the SHAP summary plot (Figure 3) reinforced the importance of features such as site of lesion, pulse, and total protein as critical predictors, consistent with findings in other equine health studies [4,7]. By combining global and local explanations, SHAP provides a robust framework for understanding survival determinants, enabling targeted and evidence-based interventions. These insights not only optimize clinical decision-making but also align with broader efforts to enhance animal welfare and veterinary care standards.

Beyond equine colic management, the implications of SHAP extend to broader animal health contexts. The method’s ability to generate interpretable insights makes it applicable to other species and conditions, offering a scalable solution for improving diagnostic and treatment strategies across veterinary medicine. For instance, machine learning frameworks have been successfully applied to predict foot-and-mouth disease outbreaks in cattle farms, leveraging environmental and management-related risk factors to inform disease control strategies [34]. Similarly, classification tree models have been utilized to identify critical risk factors for Brucella infection in dairy cattle, demonstrating superior accuracy compared to traditional statistical approaches [10]. By integrating diverse datasets and advancing interpretability through techniques like SHAP, the presented AI model could be adapted to a broader spectrum of clinical challenges, enhancing decision-making and personalized care in various domains.

### Limitations and Future Works

This study highlights the potential of AI in equine colic outcome prediction but has certain limitations. First, the dataset used, though reliable, was limited in size and diversity, particularly in terms of clinical case variety. Including a broader range of colic types, such as control, strangulating, and inflammatory cases, would strengthen the model’s robustness and generalizability. Second, the precision of the AI model, while promising, was in line with prior colic estimation models. This suggests the potential for hybrid approaches combining traditional and AI-based methods to enhance predictive performance. Another limitation is the reliance on retrospective data, which may not fully account for recent advancements in diagnostic sensitivity over the past decade. Incorporating real-time clinical data and updated diagnostic tools in future studies could address this gap. Furthermore, although SHAP was used for model interpretability, more intuitive visualizations and clinician-friendly interfaces are needed to facilitate practical adoption.

Future work will focus on expanding the dataset, integrating additional clinical variables, exploring model combinations, and validating the AI model through prospective clinical trials to ensure its relevance and usability in real-world settings. Another promising avenue is the incorporation of additional data sources, such as microbiome composition [7] or environmental variables [35], which could enhance the predictive accuracy and applicability of SHAP models in equine health management. Developing real-time decision support systems powered by XAI methods could also revolutionize veterinary care by providing clinicians with actionable insights during emergencies or critical care situations. Furthermore, conducting comparative studies across different species, geographical regions, or clinical settings would validate the generalizability of our approach and its utility in diverse veterinary contexts. These efforts would not only refine the application of XAI in animal health but also contribute to the broader goals of precision medicine and improved animal welfare.

## 5. Conclusions

In conclusion, personalized insights derived through SHAP represent the most transformative finding of this study. They empower veterinarians with actionable knowledge tailored to individual cases, bridging the gap between predictive modeling and real-world clinical application. This personalized approach to equine care has the potential to significantly enhance survival outcomes and improve overall welfare.

## Figures and Tables

**Table 1:** Machine learning models for survival predictions.

Models	Categories	Descriptions
**Random Forest (RF)**	Bagging, Decision Trees	Combines decision trees using the bagging method [16,17].
**Support Vector** **Machine (SVM)**	Kernel Functions, Hyperplanes	Separates data using linear or non-linear hyperplanes [18,19].
**Gaussian Naive** **Bayes**	Bayes Theorem, Gaussian Distribution	A classifier based on Bayes’ theorem and Gaussian distribution [20,21].
**K-Nearest** **Neighbors (KNN)**	Distance Measurements	Classifies or regresses based on distances between data points [22,23].
**XGBoost**	Gradient Boosting, Quadratic Derivatives	A fast and powerful boosting algorithm that reduces errors iteratively [24,25].
**LightGBM** **(LGBM)**	Gradient Boosting, Histogram Algorithm	Uses histogram-based data splitting for speed and memory efficiency [26,27].
**AdaBoost**	Weighted Error Minimization	Combines weak learners with weighted boosting [28,29].
**HistGradientBoost**	Gradient Boosting, Histogram Algorithm	Employs histogram-based gradient boosting for improved performance [30,31].

**Table 2:** Performance metrics of machine learning models for survival predictions.

	Models	Accuracy	Recall	Precision	F1 Score
1	Random Forest	**0.861**	0.859	0.860	0.859
2	XGBoost	0.860	**0.860**	**0.862**	**0.861**
3	HistGradientBoost	0.847	0.846	0.848	0.847
4	LGBM	0.834	0.832	0.833	0.832
5	AdaBoost	0.833	0.834	0.836	0.835
6	KNN	0.820	0.819	0.822	0.820
7	SVM	0.736	0.735	0.752	0.743
8	Gaussian Naive Bayes	0.735	0.733	0.783	0.717

## Data Availability

The data utilized in this study were acquired from an open-source database: McLeish, M.; Cecile, M. Horse Colic [Dataset]. *UCI Machine Learning Repository*. 1989. https://doi.org/10.24432/C58W23. [15]. UCI Machine Learning Repository. An alternative version of this dataset is also accessible via Kaggle: https://www.kaggle.com/datasets/yasserh/horse-survival-dataset (accessed on 10 October 2024).

## Appendix A

Table A1 provides a detailed description of the Horse Survival Dataset features after preprocessing, including their types, categorical or numerical values, and the specific transformations applied to ensure consistency and relevance for the analysis.

**Table A1:** Horse survival dataset feature descriptions after preprocessing.

Features	Feature Information	Types
Age	Adult, young (<6 months)	Categoric
Temperature of extremities	Normal, warm, cool, cold	
Peripheral pulse	Normal, increased, reduced, absent	
Mucous membranes	Normal pink, bright pink, pale pink, pale cyanotic, bright red, dark cyanotic	
Capillary refill time	<3 s, ≥3 s	
Pain	No pain, depressed, mild pain, severe pain, extreme pain	
Peristalsis	Hypermotile, normal, hypomotile, absent	
Abdominal distension	None, slight, moderate, severe	
Nasogastric tube	None, slight, significant	
Nasogastric reflux	None, >1 L, <1 L	
Rectal examination-feces	Normal, increased, decreased, absent	
Abdomen	Normal, other, firm, small intestine, large intestine	
Abdominocentesis appearance	Clear, cloudy, serosanguinous	
Surgical lesion	No: non-surgical lesion/Yes: surgical lesion	
Cp data	No: pathology data not present/Yes: data present	
Surgery	No: horse had surgery/Yes: without surgery	
site_of_lesion1	1 = gastric, 2 = sm intestine, 3 = lg colon, 4 = lg colon and cecum, 5 = cecum, 6 = transverse colon, 7 = rectum/descending colon, 8 = uterus, 9 = bladder, 11 = all intestinal sites	
type_of_lesion1	1 = simple, 2 = strangulation, 3 = inflammation, 4 = other	
subtype_of_lesion1	1 = mechanical, 2 = paralytic	
specific_code_of_lesion1	1 = obturation, 2 = intrinsic, 3 = extrinsic, 4 = adynamic, 5 = volvulus/torsion, 6 = intussusception, 7 = thromboembolic, 8 = hernia, 9 = lipoma/splenic incarceration, 10 = displacement	
lesion_2_info	Presence, absence	
lesion_3_info	Presence, absence	
Rectal temperature	Min: 35.4–Max: 40.8	Numeric
Nasogastric reflux pH	Min: 1–Max: 7.5	
Pulse	Min: 30–Max: 184	
Respiratory rate	Min: 8–Max: 96	
Packed cell volume	Min: 23–Max: 75	
Total protein	Min: 3.3–Max: 8.9	
Abdominocentesis total protein	Min: 0.1–Max: 10.1	
Outcome (Survive)	Lived, died (euthanized and died, merged as died)	Target (Categoric)

## Appendix B

### Appendix B.1. Missing Data Handling

Classical approaches were employed to handle missing data in this study, ensuring the dataset’s integrity and reliability. Missing values in categorical variables were addressed using the mode, which represents the most frequently occurring value, as it maintains the distribution of the data. For numerical variables, measures of central tendency such as the mean or median were used based on the data’s distribution characteristics. For instance, variables with a normal distribution were filled with the mean, while those with skewed distributions were imputed using the median. These methods were chosen to preserve the underlying patterns within the dataset and minimize the risk of introducing bias or distorting the relationships between features. By applying these targeted imputation strategies, the dataset was prepared to support robust and accurate machine learning model development.

### Appendix B.2. Label Encoding

We implemented feature engineering techniques to optimize the performance of our machine learning models for predicting horse survival. The dataset included a mix of categorical and numerical features, all of which were carefully processed to extract valuable insights. Categorical variables such as “Surgery”, “Age”, “MucousMembrane”, “PainLevel”, and “AbdominalDistention” were transformed using label encoding. This technique assigns a unique numeric value to each category, enabling the categorical features to be effectively utilized by machine learning models. Additionally, the “HospitalNumber” column, which served as a unique identifier without predictive value, was removed to simplify the dataset. Numerical features such as “RectalTemperature”, “Pulse”, “PackedCellVolume”, and “RespiratoryRate” were retained in their original form and used directly in model training. These features were identified as key predictors of survival during exploratory data analysis. The engineered features provide a robust foundation for model development, ensuring the models effectively capture the relationships between the input variables and the target outcome of horse survivability.

### Appendix B.3. Decoding of “Lesion” Features

The data in the ‘Lesion_1’, ‘Lesion_2’, ‘Lesion_3’ features in the dataset are provided in coded form. Although at first glance these featurettes appear numeric, they contain categorical information. The data in these features represent different information according to their steps. The numbers in these features contain the following information according to their digits, respectively.

**Table A2:** Original coding descriptions of “lesion” features. N/A—not available.

Site of Lesion	Type	Subtype	Specific Code
1 = Gastric, 2 = Small Intestine, 3 = Large Colon, 4 = Large Colon and Cecum, 5 = Cecum, 6 = Transverse Colon, 7 = Rectum/Descending Colon, 8 = Uterus, 9 = Bladder, 11 = All Intestinal Sites, 00 = None,	1 = Simple, 2 = Strangulation, 3 = Inflammation, 4 = Other	1 = Mechanical, 2 = Paralytic, 0 = N/A	1 = Obstruction, 2 = Intrinsic, 3 = Extrinsic, 4 = Adynamic, 5 = Volvulus/Torsion, 6 = Intussusception, 7 = Thromboembolic, 8 = Hernia, 9 = Lipoma/Splenic Incarceration 10 = Displacement, 0 = N/A

Different features were created using this information. These are site_of_lesion1, type_of_lesion1, subtype_of_lesion1, specific_code_of_lesion1, lesion_2_info, lesion_3_info. The lesion_1 feature provides the most information; the lesion_2 and lesion_3 features provide relatively little information, so only their presence or absence was analyzed.

### Appendix B.4. Evaluation Metrics

Each model was evaluated based on accuracy, precision, recall, and F1 scores to determine the best-performing algorithm as follows:

**Table A3:** Evaluation Metrics.

Metric	Formula
**Accuracy:** The ratio of correctly predicted observations to the total observations. It is useful when the dataset is balanced.	$\frac{True\;Positives+True\;Negatives}{Total\;Instances}\;\;$
**Precision:** The ratio of correctly predicted positive observations to the total predicted positive observations. Focuses on how many selected items are relevant.	$\frac{True\;Positives}{True\;Positives+False\;Negatives}$
**Recall:** The ratio of correctly predicted positive observations to all actual positives. Also known as sensitivity.	$\frac{True\;Positives}{True\;Positives+False\;Positives}\;\;\;$
**F1 Score:** The harmonic mean of precision and recall. It is more suitable when the dataset has imbalanced classes, balancing precision and recall.	$2\times \frac{Precision\times Recall}{Precision+Recall}\;\;\;\;\;$
